# Construction of the prognostic model for small‐cell lung cancer based on inflammatory markers: A real‐world study of 612 cases with eastern cooperative oncology group performance score 0–1

**DOI:** 10.1002/cam4.5728

**Published:** 2023-04-04

**Authors:** Chang Liu, Bo Jin, Yunpeng Liu, Ouyang Juhua, Bowen Bao, Bowen Yang, Xiuming Liu, Ping Yu, Ying Luo, Shuo Wang, Zan Teng, Na Song, Jinglei Qu, Jia Zhao, Ying Chen, Xiujuan Qu, Lingyun Zhang

**Affiliations:** ^1^ Department of Medical Oncology The First Hospital of China Medical University Shenyang China; ^2^ Key Laboratory of Anticancer Drugs and Biotherapy of Liaoning Province The First Hospital of China Medical University Shenyang China; ^3^ Liaoning Province Clinical Research Center for Cancer Shenyang China

**Keywords:** inflammatory markers, nomogram, prognostic factors, small‐cell lung cancer, survival

## Abstract

**Objectives:**

This research aimed to explore the relationship between pre‐treatment inflammatory markers and other clinical characteristics and the survival of small‐cell lung cancer (SCLC) patients who received first‐line platinum‐based treatment and to construct nomograms for predicting overall survival (OS) and progression‐free survival (PFS).

**Methods:**

A total of 612 patients diagnosed with SCLC between March 2008 and August 2021 were randomly divided into two cohorts: a training cohort (*n* = 459) and a validation cohort (*n* = 153). Inflammatory markers, clinicopathological factors, and follow‐up information of patients were collected for each case. Cox regression was used to conduct univariate and multivariate analyses and the independent prognostic factors were adopted to develop the nomograms. Harrell's concordance index (C‐index) and time‐dependent receiver operating characteristic curve were used to verify model differentiation, calibration curve was used to verify consistency, and decision curve analysis was used to verify the clinical application value.

**Results:**

Our results showed that baseline C‐reactive protein/albumin ratio, neutrophil/lymphocyte ratio, NSE level, hyponatremia, the efficacy of first‐line chemotherapy, and stage were independent prognostic factors for both OS and PFS in SCLC. In the training cohort, the C‐index of PFS and OS was 0.698 and 0.666, respectively. In the validation cohort, the C‐index of PFS and OS was 0.727 and 0.747, respectively. The nomograms showed good predictability and high clinical value. Also, our new clinical models were superior to the US Veterans Administration Lung Study Group (VALG) staging for predicting the prognosis of SCLC.

**Conclusions:**

The two prognostic nomograms of SCLC including inflammatory markers, VALG stage, and other clinicopathological factors had good predictive value and could individually assess the survival of patients.

## Abstract 2

We explored the relationship between pre‐treatment inflammatory markers and other clinical characteristics and the survival of small‐cell lung cancer (SCLC) patients who received first‐line platinum‐based treatment. The nomograms for predicting progression‐free survival and overall survival had good predictability and stability. This research may provide clues and guides for individual treatment of SCLC patients.

## 1 INTRODUCTION

Small‐cell lung cancer (SCLC) is a malignant neuroendocrine tumor accounting for about 15%–20% of all types of lung cancers.^1^ SCLC has adverse biological behaviors, including a high proliferation index, short doubling time, strong invasive ability, and susceptibility to metastasis in the early stage of the disease.^2^ Two‐thirds of SCLC patients present with distant metastases and the remaining patients who initially present with limited‐stage disease will easily develop into metastatic disease and chemoresistance. The median overall survival (OS) has previously been reported to be 8–13 months and 15–20 months in the extensive stage SCLC (ES‐SCLC) and limited‐stage SCLC (LS‐SCLC), respectively.^3^ However, according to recent studies, the additional use of immunotherapeutic agents durvalumab or atezolizumab in the first‐line treatment of ES‐SCLC could promote a survival improvement of only 2–3 months,^4^, ^5^ and the ASTRUM005 Phase 3 trial for ES‐SCLC showed a remarkable 4.5‐month additional survival benefit.^6^

Recent studies have shown that SCLC is a heterogeneous disease based on transcription factor typing,^7^ but molecular typing is not yet widely used. It is necessary to find appropriate individual stratified variables to better guide the treatment of SCLC patients. Some prognostic factors of SCLC were reported such as: clinical stage, neuron‐specific enolase (NSE) level, hyponatremia, lactate dehydrogenase level (LDH), and initial chemotherapy efficacy.^8^, ^9^, ^10^, ^11^, ^12^ Recent studies show that systemic inflammation also plays a key role in determining clinical outcomes of SCLC patients.^13^, ^14^ Previous studies reported some serum systemic inflammatory markers such as C‐reactive protein (CRP)/albumin ratio (CAR), albumin/globulin ratio (AGR), neutrophil/lymphocyte ratio (NLR), platelet/lymphocyte ratio (PLR), and prognostic nutritional index (PNI) may affect the survival of SCLC.^15^, ^16^, ^17^ Other inflammatory markers such as lymphocyte/CRP ratio (LCR) has however, never been reported in SCLC and whether lymphocyte/monocyte ratio (LMR) and red blood cell distribution width (RDW) can predict the survival of SCLC remain controversial.^18^, ^19^, ^20^, ^21^, ^22^

In this real‐world study, we retrospectively investigated survival information, pre‐treatment inflammatory markers and other clinicopathological characteristics of 612 SCLC cases from northeast China. By collecting indicators that may be associated with SCLC, we developed two nomograms for predicting the OS and PFS of SCLC patients. Inflammatory markers that are clinically feasible and convenient to distinguish were determined to predict the prognosis of SCLC patients. The research may provide clues for individual treatment of SCLC patients.

## 2 MATERIALS AND METHODS

### 2.1 Data collection

A total of 612 patients pathologically diagnosed with SCLC from the First Hospital of China Medical University between March 2008 and August 2021 were included. Inclusion criteria were as follows: availability of complete patient records; no previous tumor‐related treatment; patients who received at least two cycles of standard first‐line platinum‐based doublet chemotherapy and at least once standardized evaluation of efficacy; no previous history of other primary tumors except for SCLC; Eastern Cooperative Oncology Group (ECOG) performance score 0–1. There were only 22 patients receiving PD‐L1 inhibitors in combination with platinum‐based chemotherapy in the first‐line treatment. The last follow‐up date was May 1, 2022. OS was defined as the interval from the date of initial diagnosis to death. OS time for patients who lost to follow‐up or were alive as of last follow‐up was equal to the date of last follow‐up. PFS was the time interval from original diagnosis to documented tumor progression or death due to any cause.

### 2.2 Biomarkers and other variables

SCLC baseline clinicopathological factors were collected for each subject, such as gender, age, smoking history, US Veterans Lung Cancer Society (VALG) staging, chemotherapy, and thoracic radiotherapy history. Also, pre‐treatment lymphocyte, neutrophils, platelet, hemoglobin, RDW, albumin, globulin, total protein, LDH, carcino‐embryonic antigen (CEA), NSE, CRP, and blood sodium were recorded and evaluated by the hospital's examination criteria. All patients underwent standardized evaluation of the best efficacy of first‐line chemotherapy. NLR, PLR, AGR, LMR, CAR, PNI, and LCR were calculated as followed:

$$\mathrm{NLR}=\text{neutrophil count}\;\left(10^{9}/L\right)/\text{lymphocyte count}\;\left(10^{9}/L\right)$$

$$\mathrm{PLR}=\text{platelet count}\;\left(10^{9}/L\right)/\text{lymphocyte count}\;\left(10^{9}/L\right)$$

$$\mathrm{AGR}=\text{albumin}\;\left(g/L\right)/\left(\text{total protein}-\text{albumin}\right)\;\left(g/L\right)$$

$$\mathrm{CAR}=\mathrm{CRP}\;\left(\mathrm{mg}/L\right)/\text{albumin}\;\left(g/L\right)$$

$$\mathrm{PNI}=10\times \text{albumin}\;\left(g/L\right)+0.005\times \text{lymphocyte count}\;\left(10^{9}/L\right)$$

$$\mathrm{LCR}=\text{lymphocyte count}\;\left(10^{9}/L\right)/\mathrm{CRP}\;\left(\mathrm{mg}/L\right)$$

$$\mathrm{LMR}=\text{lymphocyte count}\;\left(10^{9}/L\right)/\text{monocyte}\;\left(10^{9}/L\right).$$

### 2.3 Statistical analysis

#### 2.3.1 Nomogram development and construction

SPSS 25.0 and R 4.0.5 were used to conduct data analysis and caret R package was used to divide the training cohort and validation cohort. Pearson correlation coefficient was tested to ensure that there was no potential collinearity and interaction between each variable. The cut‐off level of NLR, PLR, AGR, CAR, PNI, LCR, and LMR was calculated by the survival and survminer R package. The above continuous variables were transformed into binary variables according to the cut‐off value. Chi‐square test was used to compare the clinicopathological parameters among the training cohort and the validation cohort. Cox's proportional hazards model was used to conduct univariate and multivariate survival analyses of OS and PFS. Factors with a *p*‐value <0.1 in univariate analysis were included in multivariate Cox regression for independent prognostic analysis. Statistically independent variables (*p* < 0.05) in multivariate analysis were used to construct nomogram plots of 8‐, 12‐, and 24‐month probability for OS and 6‐, 12‐, and 18‐month probability for PFS.

#### 2.3.2 Nomogram validation

Harrell's concordance index (C‐index) and time‐dependent receiver operating characteristic (ROC) curve were used to verify the model differentiation.^23^, ^24^, ^25^ A bootstrap method (number of self‐sampling operations B was 1000) was used for internal validation and plotting calibration curves to verify consistency. Decision curve analysis (DCA) was used to estimate the value of the nomogram in terms of competing benefits and problems. The horizontal line represents that all samples do not receive the intervention and the return rate is 0, the slash line represents that all samples receive the intervention, and the solid line represents the net income of the nomogram models.

## 3 RESULTS

### 3.1 Patient characteristics

**FIGURE 1:**
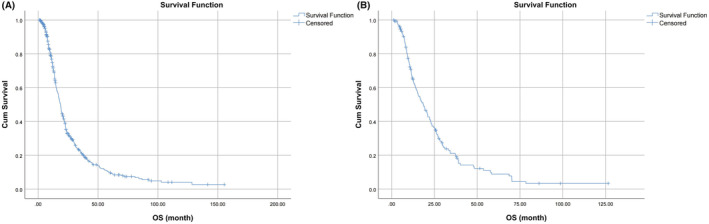
OS curves of SCLC in the training (A) and validation cohort (B).

In accordance with the inclusion criteria of this study, a total of 612 patients were randomly split into two cohorts, a training cohort (*n* = 459) and an independent internal validation cohort (*n* = 153), at a 3:1 ratio. Table 1 lists the characteristics of patients across the two cohorts. No significant differences in the characteristics were observed between the two groups. The median OS for training and validation cohort was 18.67 months [95% confidence interval (CI), 17.31–20.03], and 18.53 months (95% CI, 14.49–22.58), respectively (Figure 1). The median follow‐up time was 67.6 months. The rate of patients who died at the time of last follow‐up was 77.1% in the training cohort and 79% in the validation cohort, respectively. Further analysis showed the median OS was 12 months in ES‐SCLC and 19.28 months LS‐SCLC, respectively.

**TABLE 1:** Comparison of baseline characteristics between training and validation cohorts of SCLC.

Characteristics	Training cohort (%)	Validation cohort (%)	*χ* ^2^	*p* value
	(*N* = 459)	(*N* = 153)		
Sex				
Male	336 (73.2%)	109 (71.2%)	0.222	0.637
Female	123 (26.8%)	44 (28.8%)		
Age				
≤65	332 (72.3%)	118 (77.1%)	1.354	0.245
>65	127 (27.7%)	35 (22.9%)		
Smoking history				
Yes	284 (61.9%)	95 (62.1%)	0.002	0.962
No	175 (38.1%)	58 (37.9%)		
Thoracic radiotherapy				
Yes	277 (60.3%)	90 (58.8%)	0.111	0.739
No	182 (39.7%)	63 (41.2%)		
Four cycle standard platinum‐based chemotherapy				
Yes	396 (86.3%)	131 (85.6%)	0.041	0.840
No	63 (13.7%)	22 (14.4%)		
The best efficacy to first‐line chemotherapy				
CR + PR	320 (69.7%)	108 (70.6%)	0.041	0.839
SD + PD	139 (30.3%)	45 (29.4%)		
Brain metastasis				
Yes	41 (8.9%)	14 (9.2%)	0.007	0.935
No	418 (91.1%)	139 (90.8%)		
Liver metastasis				
Yes	70 (15.3%)	24 (15.7%)	0.017	0.897
No	389 (84.7%)	129 (84.3%)		
Bone metastasis				
Yes	54 (11.8%)	18 (11.8%)	0.001	1.000
No	405 (88.2%)	135 (88.2%)		
Stage				
ES‐SCLC	207 (45.1%)	75 (49.0%)	0.710	0.399
LS‐SCLC	252 (54.9%)	78 (51.0%)		
CAR				
>0.51	70 (15.3%)	29 (19.0%)	1.161	0.281
≤0.51	389 (84.7%)	124 (81.0%)		
NLR				
>1.28	414 (90.2%)	145 (94.8%)	3.037	0.081
≤1.28	45 (9.8%)	8 (5.2%)		
AGR				
<1.36	127 (27.7%)	43 (28.1%)	0.011	0.917
≥1.36	332 (72.3%)	110 (71.9%)		
RDW				
<14.3	414 (90.2%)	141 (92.2%)	0.522	0.470
≥14.3	45 (9.8%)	12 (7.8%)		
PLR				
>118.8	304 (66.2%)	112 (73.2%)	2.562	0.109
≤118.8	155 (33.8%)	41 (26.8%)		
LMR				
<4.59	336 (73.2%)	119 (77.8%)	1.259	0.262
≥4.59	123 (26.8%)	34 (22.2%)		
LCR				
<0.39	194 (42.3%)	62 (40.5%)	0.143	0.705
≥0.39	265 (57.7%)	91 (59.5%)		
PNI				
<397.0	188 (41.0%)	65 (42.5%)	2.520	0.112
≥397.0	271 (59.0%)	88 (57.5%)		
Hyponatremia				
Yes	66 (14.4%)	26 (17.0%)	0.614	0.433
No	393 (85.6%)	127 (83.0%)		
LDH (U/L)				
>250	145 (31.6%)	40 (26.1%)	1.614	0.204
≤250	314 (68.4%)	113 (73.9%)		
Hemoglobin (g/L)				
<120	86 (18.7%)	30 (19.6%)	0.057	0.812
≥120	373 (81.3%)	123 (80.4%)		
CEA (ng/mL)				
>4.3	171 (37.3%)	47 (30.7%)	2.138	0.144
≤4.3	288 (62.7%)	106 (69.3%)		
NSE (ng/mL)				
>16.3	393 (85.6%)	140 (91.5%)	3.532	0.060
≤16.3	66 (14.4%)	13 (8.5%)		

### 3.2 Survival analysis

#### 3.2.1 Prognostic analysis of OS for SCLC patients

As shown in Table 2, univariate analysis found that 15 pre‐treatment parameters were negatively associated with OS. Multivariate analysis showed seven factors were independent risk factors for OS: baseline CAR > 0.51 (HR = 1.615, *p* = 0.001), NLR > 1.28 (HR = 1.705, *p* = 0.004), AGR < 1.36 (HR = 1.337, *p* = 0.018), NSE > 16.3 ng/mL (HR = 1.656, *p* = 0.004), hyponatremia (HR = 1.544, *p* = 0.003), SD + PD for the best efficacy to first‐line chemotherapy (HR = 1.641, *p* < 0.001), and extensive stage (HR = 1.731, *p* < 0.001).

**TABLE 2:** COX regression analysis of clinical characteristics in connection with OS in the training cohort of SCLC.

Risk factors	Univariate analysis		Multivariate analysis	
	HR (95%CI)	*p* Value	HR (95%CI)	*p* Value
CAR (>0.51 vs. ≤0.51)	1.974 (1.510–2.580)	<0.001	1.615 (1.211–2.153)	0.001
NLR (>1.28 vs. ≤1.28)	1.773 (1.242–2.531)	0.002	1.705 (1.181–2.461)	0.004
AGR (<1.36 vs. ≥1.36)	1.600 (1.278–2.003)	<0.001	1.337 (1.051–1.701)	0.018
NSE (ng/mL) (>16.3 vs. ≤16.3)	1.902 (1.359–2.662)	<0.001	1.656 (1.179–2.326)	0.004
Hyponatremia (yes vs. no)	1.563 (1.179–2.074)	0.002	1.544 (1.155–2.063)	0.003
The best efficacy to first‐line chemotherapy (SD + PD vs. CR + PR)	1.664 (1.331–2.080)	<0.001	1.641 (1.308–2.058)	<0.001
Stage (ES‐SCLC vs. LS‐SCLC)	1.777 (1.441–2.192)	<0.001	1.731 (1.395–2.148)	<0.001
LDH (U/L) (>250 vs. ≤250)	1.439 (1.153–1.797)	0.001		
Hemoglobin (g/L) (<120 vs. ≥120)	1.510 (1.163–1.960)	0.002		
Age (>65 vs. ≤65)	1.321 (1.045–1.670)	0.020		
Sex (male vs. female)	1.233 (0.973–1.562)	0.083		
Smoking history (Yes vs. No)	1.180 (0.951–1.463)	0.132		
RDW (<14.3 vs. ≥14.3)	0.819 (0.545–1.231)	0.338		
PLR (>118.8 vs. ≤118.8)	1.509 (1.204–1.892)	<0.001		
LMR (<4.59 vs. ≥4.59)	1.607 (1.253–2.061)	<0.001		
PNI (<397.0 vs. ≥397.0)	1.517 (1.229–1.872)	<0.001		
CEA (ng/mL) (>4.3 vs. ≤4.3)	1.326 (1.069–1.643)	0.010		
LCR (<0.39 vs. ≥0.39)	1.613 (1.299–2.001)	<0.001		

#### 3.2.2 Prognostic analysis of PFS for SCLC patients

As displayed in Table 3, univariate analysis found that 14 pre‐treatment parameters were negatively related to PFS. The final multivariate analysis showed seven independent negative markers for PFS: baseline CAR > 0.51 (HR = 1.643, *p* = 0.003), NLR > 1.28 (HR = 2.017, *p* = 0.002), LDH > 250 U/L (HR = 1.345, *p* = 0.027), NSE > 16.3 ng/mL (HR = 1.827, *p* = 0.003), hyponatremia (HR = 1.483, *p* = 0.017), SD + PD for the best efficacy to first‐line chemotherapy (HR = 2.045, *p* < 0.001), and extensive stage (HR = 2.146, *p* < 0.001) .

**TABLE 3:** Cox regression analysis of clinical characteristics in connection with PFS in the training cohort of SCLC.

Risk factors	Univariate analysis		Multivariate analysis	
	HR (95%CI)	*p* Value	HR (95%CI)	*p* Value
CAR (>0.51 vs. ≤0.51)	1.890 (1.370–2.607)	<0.001	1.643 (1.182–2.283)	0.003
NLR (>1.28 vs. ≤1.28)	2.205 (1.431–3.397)	<0.001	2.017 (1.292–3.147)	0.002
LDH (U/L) (>250 vs. ≤250)	1.619 (1.257–2.086)	<0.001	1.345 (1.034–1.750)	0.027
NSE (ng/mL) (>16.3 vs. ≤16.3)	1.895 (1.287–2.789)	0.001	1.827 (1.222–2.732)	0.003
Hyponatremia (yes vs. no)	1.549 (1.133–2.119)	0.006	1.483 (1.073–2.049)	0.017
The best efficacy to first‐line chemotherapy (SD + PD vs. CR + PR)	2.075 (1.601–2.690)	<0.001	2.045 (1.569–2.665)	<0.001
Stage (ES‐SCLC vs. LS‐SCLC)	2.146 (1.682–2.737)	<0.001	2.146 (1.663–2.770)	<0.001
AGR (<1.36 vs. ≥1.36)	1.504 (1.145–1.974)	0.003		
Hemoglobin (g/L) (<120 vs. ≥120)	1.501 (1.101–2.045)	0.010		
Age (>65 vs. ≤65)	1.074 (0.808–1.428)	0.623		
Sex (male vs. female)	1.127 (0.862–1.474)	0.382		
Smoking history (Yes vs. No)	1.194 (0.938–1.521)	0.150		
RDW (<14.3 vs. ≥14.3)	0.884 (0.413–1.894)	0.751		
PLR (>118.8 vs. ≤118.8)	1.501 (1.160–1.940)	0.002		
LMR (<4.59 vs. ≥4.59)	1.455 (1.105–1.915)	0.007		
PNI (<397.0 vs. ≥397.0)	1.281 (1.003–1.635)	0.047		
CEA (ng/mL) (>4.3 vs. ≤4.3)	1.318 (1.023–1.699)	0.033		
LCR (<0.39 vs. ≥0.39)	1.693 (1.321–2.170)	<0.001		

### 3.3 Construction of predictive nomograms and evaluation of the predictive effect

#### 3.3.1 Nomograms development

**FIGURE 2:**
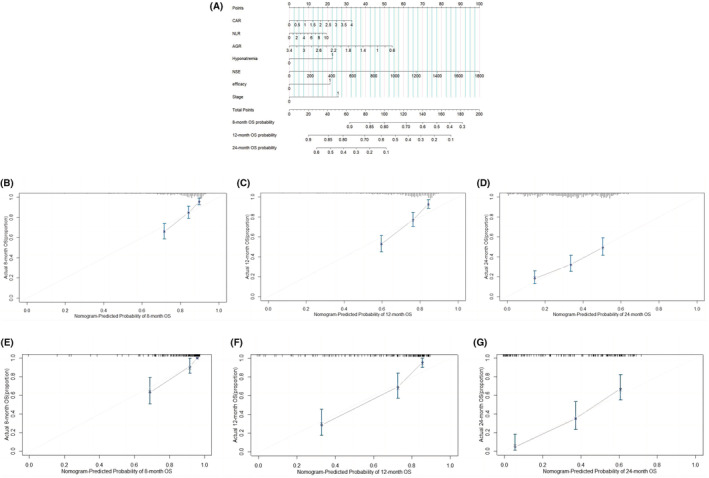
(A) Nomogram predicted 8‐, 12‐, and 24‐month OS for SCLC patients. The total points predicted on the bottom scale represent the probability of 8‐, 12‐, and 24‐month survival rates. (B–D) Calibration curves for predicting 8‐(B), 12‐(C), and 24‐month (D) OS of SCLC patients in the training cohort. The X‐axis denotes survival predicted by the nomogram and the Y‐axis represents actual OS as measured by the Kaplan–Meier analysis. (E–G) Calibration curves for predicting 8‐(E), 12‐(F), and 24‐month (G) OS of SCLC patients in the validation cohort.

**FIGURE 3:**
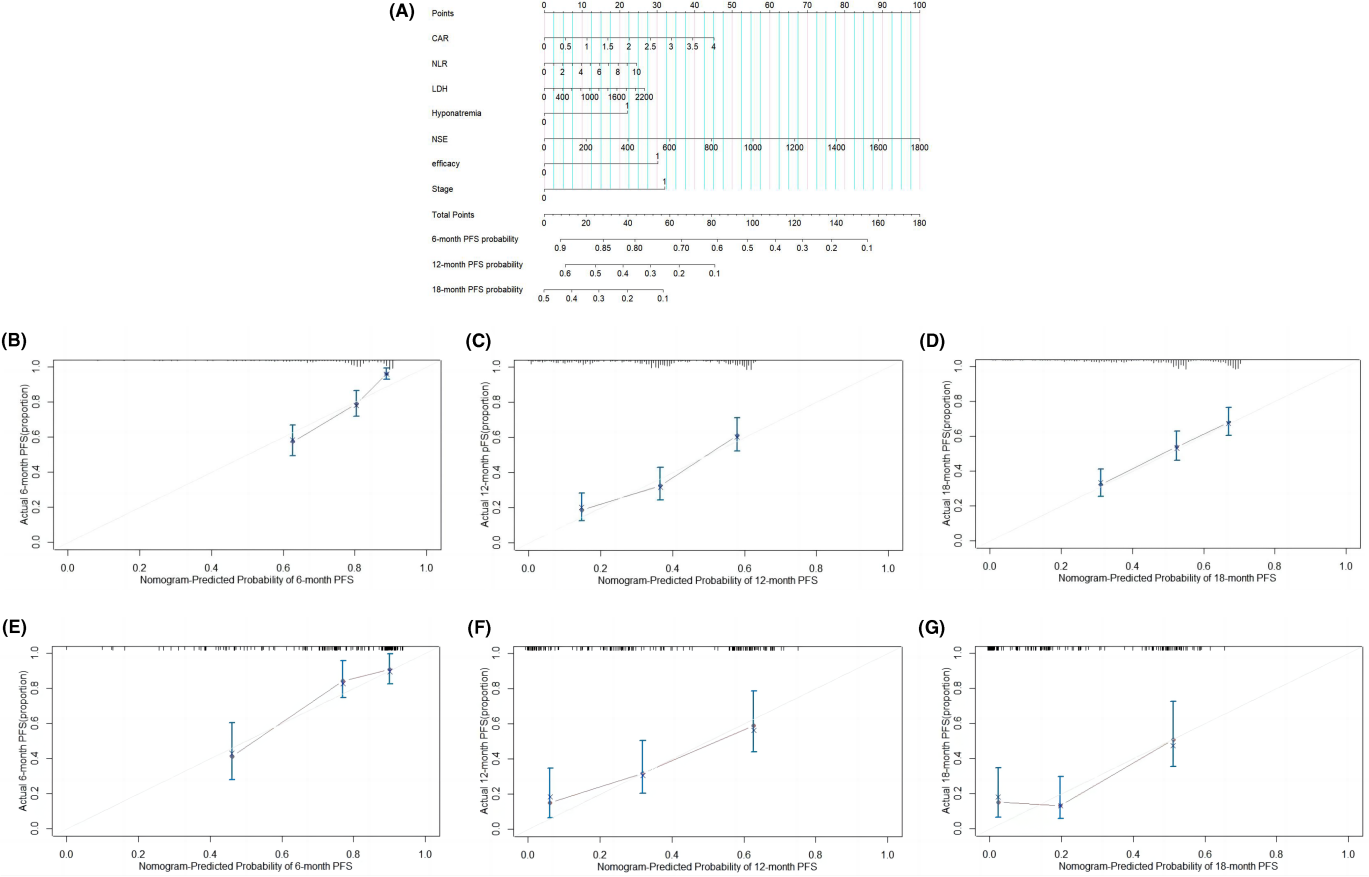
(A) Nomogram predicted 6‐, 12‐, and 18‐month PFS for SCLC patients. (B–D) Calibration curves for predicting 6‐(B), 12‐(C), and 18‐month (D) PFS of SCLC patients in the training cohort. (E–G) Calibration curves for predicting 6‐(E), 12‐(F), and 18‐month (G) PFS of SCLC patients in the validation cohort.

The nomograms predicting OS (Figure 2A) and PFS (Figure 3A) in SCLC patients were constructed based on the Cox regression results. Outcomes were reported as 8‐, 12‐, and 24‐month OS probability and 6‐, 12‐, and 18‐month PFS probability, respectively. The nomograms assigned points based on CAR, NLR, AGR, LDH, and NSE in a continuous but nonlinear way. In the nomogram models, projecting upward to a small scale according to different categories of each feature can obtain the score of each item. Adding the scores of each item to the total score and projecting downward from the total scale can obtain the survival rates.

#### 3.3.2 Nomograms validation

In the training cohort, the bootstrap C‐index of the nomogram was 0.666 (95% CI: 0.635–0.697) and 0.698 (95% CI: 0.665–0.731) of OS and PFS, respectively. Similar results were shown in the validation set, with C‐index 0.747 (95% CI: 0.708–0.786) and 0.727 (95% CI: 0.664–0.790) of OS and PFS, respectively. The calibration curves of 8, 12, and 24‐month OS and 6, 12, and 18‐month PFS probabilities in both training cohorts (Figures 2B–D and 3B–D) and validation cohorts showed good consistency between model predictions and actual values (Figures 2E–G and 3E–G). The C‐index of the OS nomogram (0.666 ± 0.031) was also significantly higher than that of the eighth edition TNM staging system (0.550 ± 0.003, *p* < 0.001) and VALG staging system (0.539 ± 0.002, *p* < 0.001),^26^ indicating that our proposed nomogram had strong prognostic power.

**FIGURE 4:**
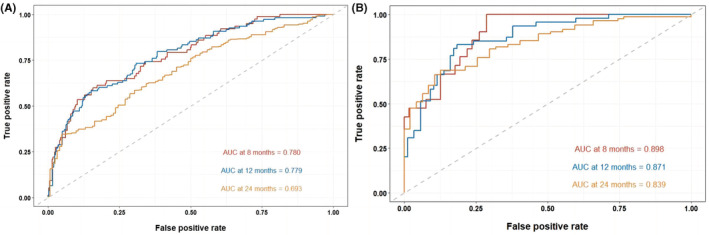
Time‐dependent ROC curves of OS at 8‐, 12‐, and 24‐month in the training cohort (A) and validation cohort (B).

**FIGURE 5:**
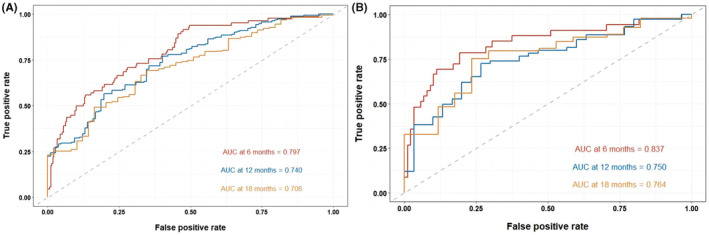
Time‐dependent ROC curves of PFS at 6‐, 12‐, and 18‐month in the training cohort (A) and validation cohort (B).

**FIGURE 6:**
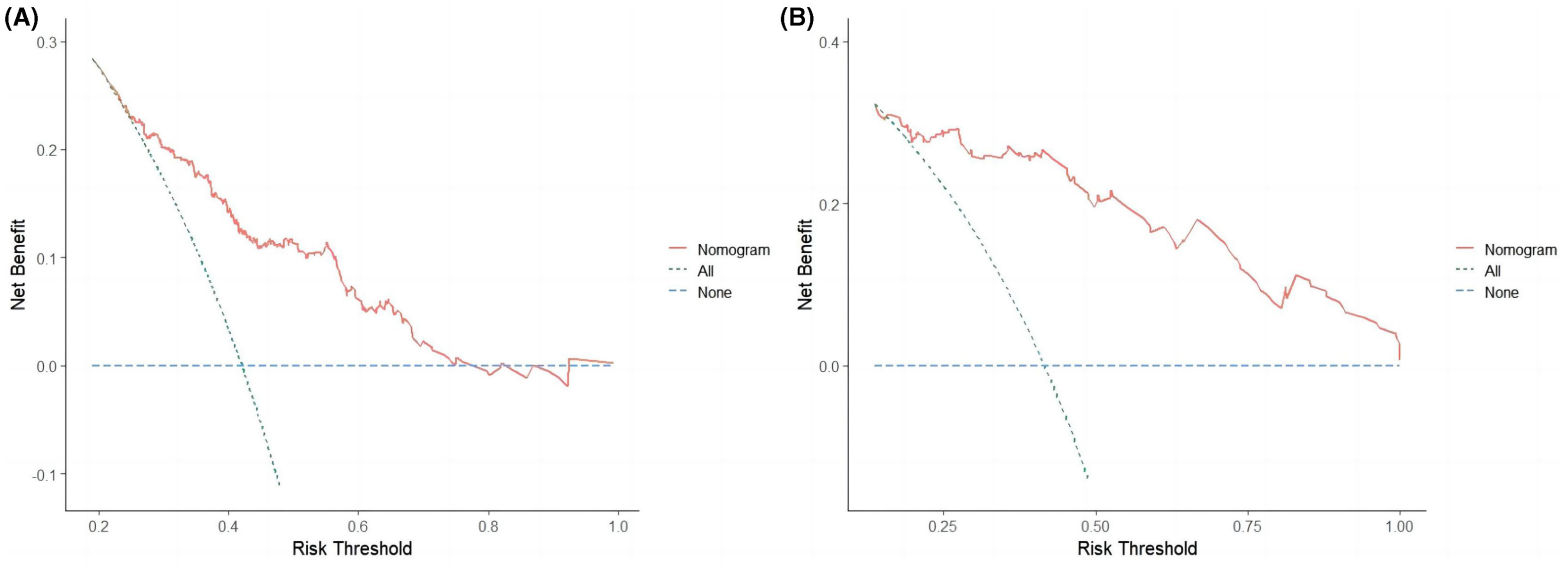
The decision curve analysis (DCA) of the OS nomogram in the training cohort (A) and validation cohort (B).

**FIGURE 7:**
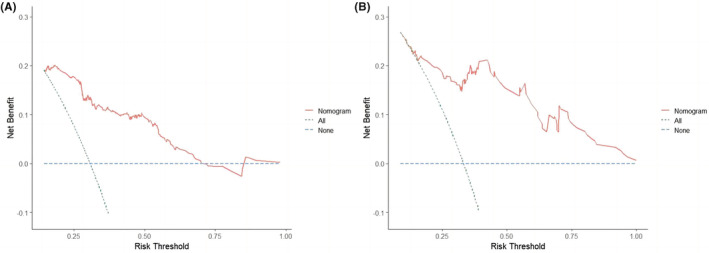
DCA of the PFS nomogram in the training cohort (A) and validation cohort (B).

In the training cohort, time‐dependent ROC curves showed the area under the curve (AUC) value in predicting 8‐, 12‐, and 24‐month OS was 0.780, 0.779, and 0.693, respectively (Figure 4A). The AUC value in predicting 6‐, 12‐, and 18‐month PFS was 0.797, 0.740, and 0.708, respectively (Figure 5A). In the validation cohort, the AUC value in predicting 8‐, 12‐, and 24‐month OS was 0.898, 0.871, and 0.839, respectively (Figure 4B). The AUC value in predicting 6‐, 12‐, and 18‐month PFS was 0.837, 0.750, and 0.764, respectively (Figure 5B). The AUC results showed high predictive value of the prognostic nomograms. Using DCA to evaluate the clinical application value of the models and the net income of the nomograms were relatively accurate. The results showed that the nomogram models had high clinical value in predicting OS (Figure 6) and PFS (Figure 7) of SCLC patients.

## 4 DISCUSSION

We developed and internally validated two nomograms for predicting OS and PFS of SCLC patients based on inflammatory markers and other clinicopathological variables. To the best of our knowledge, this was a real‐world study of maximum sample size using nomograms, ROC and DCA to ascertain the prognostic factors of Chinese SCLC patients. Also, the two prognostic nomogram models were the first to include multiple inflammatory biomarkers in SCLC and showed good predictive abilities. Recent studies indicated that, except for some traditional clinical prognostic factors, inflammation could also be critical in the initiation and progression of tumors.^17^, ^27^ CRP, a prototype acute phase protein synthesized in hepatocytes in response to inflammatory changes, has been widely reported to be associated with poorer prognosis in various types of cancer. The underlying mechanisms were as follows: tumor antigens activate the immune response; tumor growth induces tissue inflammation; tumor cells secrete inflammatory cytokines that directly or indirectly increase the CRP level.^28^, ^29^ Albumin is a safe and immunogenic protein, and low albumin suggests a malnourished state and a state of persistent systemic inflammation. Albumin has been approved to play an inhibitory role in systemic inflammation and cause the tumor progression.^30^

But the CRP/ALB ratio (CAR), a novel biomarker, can reflect both systemic inflammation and nutritional status, and calculate using the above two continuous variables, which can reduce the possibility of overestimation or underestimation and stratifies patients to more accurately predict survival. CAR has been proved to be a significant and convenient factor in predicting the short survival of patients with hematological malignancies and solid cancer.^21^, ^31^, ^32^ Zhou^16^, ^17^ suggested that CAR could independently predict OS of SCLC. We further found that high CAR was an independent negative prognostic factor for both PFS and OS in SCLC patients. As one of the main cortisol‐binding proteins, globulin would increase with the accumulation of acute phase proteins and participated in immune and inflammatory responses. It was reported that AGR can serve as an independent prognostic indicator for non‐small cell lung cancer (NSCLC), lymphoma, and gastric cancer.^33^, ^34^, ^35^ Zhou^36^ showed that AGR was an independent negative factor for predicting OS of SCLC patients (our study showed a similar result). Meanwhile, we also found AGR was significant in the univariate analysis of PFS. By further analyzing in LS‐SCLC patients, we found CAR and AGR remained independent prognostic factors for OS. As readily‐available and easily‐measurable biomarkers, combination of the CAR with AGR should be used together to predict survival more accurately in SCLC patients.

Immune cells can easily affect the occurrence and development of cancer. NLR and PLR are also markers indicative of inflammation and immune status, and have been reported to be closely related to the efficacy of immune checkpoint inhibitors in non small‐cell lung cancer (NSCLC) patients.^37^, ^38^ PD‐L1 antibody testing or TMB testing is expensive and has a long waiting time, so it is important to evaluate other readily‐available and cheap prognostic markers to initially identify SCLC patients who may benefit from immunotherapy. However, the specific relationship between NLR and PLR and the prognosis of SCLC remain controversial. A study of 299 SCLC patients showed that high preoperative NLR and PLR indicated poorer OS.^15^ Xie's^39^ study found that high NLR was an independent risk factor in ES‐SCLC and high PLR indicated a poor prognosis in LS‐SCLC, but He's^11^ study which included 234 SCLC cases showed that pre‐treatment PLR and NLR were not independent prognostic factors for OS in advanced SCLC. Our study showed that baseline high NLR was an independent negative indicator for both OS and PFS in SCLC patients. PLR was significant in the univariate analysis of PFS and OS. Further subgroup analysis suggested NLR was an independent prognostic factor for LS‐SCLC. As a cheap and convenient biomarker, NLR is significant for clinicians to evaluate SCLC patients who could benefit from immunotherapy initially.

In the last step of the aerobic glycolysis process of malignant tumor cells, LDH is responsible for the catabolism of pyruvate into lactic acid, so the level of LDH symbolizes the activity of tumor cells.^40^, ^41^ Related studies have shown that LDH may be a predictor of brain metastasis and bone metastasis in SCLC patients.^42^, ^43^ Our study showed LDH was an independent prognostic factors for PFS. SCLC patients with high baseline LDH were more likely to progress. By analyzing the data of ES‐SCLC patients further, we found these patients tended to have higher pre‐treatment LDH levels, and LDH was an independent prognostic factors for poorer OS (*p* = 0.001, HR = 1.543 (95%CI: 1.181–2.016)) and PFS (*p* < 0.001, HR = 1.718 (95%CI: 1.267–2.329)) in the SCLC patients with liver metastases. Combining serum LDH levels with other indicators for assessing the risk stratification and prognosis of SCLC is essential.

Inflammatory markers such as lymphocyte/CRP ratio (LCR) have never been reported in predicting survival in SCLC. Our study showed LCR was only significant in the univariate analysis of OS and PFS. Other factors such as LMR and RDW were not significant prognostic factors in SCLC. Consistent with previous studies, hyponatremia, extensive stage, SD + PD for the best efficacy of first‐line chemotherapy and high NSE level were independently negative prognostic factors both in OS and PFS in our present study. In our study, cut‐off values of the above inflammatory markers were calculated using the survival and survminer packages in R software, while other studies may determine cut‐off values by using X‐tile, ROC curves, or log‐rank statistics, which may lead to different results. In addition, because there will be differences in factors such as region and ethnicity of the included population, and the sample size differs, which may also cause different results between these inflammatory markers.

Compared with Xie's and Zhou's studies, we included not only some known prognostic factors of SCLC, such as the level of LDH, NSE, and CEA, but also other inflammatory factors to build more comprehensive nomograms both for OS and PFS. Also, we conducted AUC to determine the discrimination of the nomograms and used DCA to estimate the value of the nomograms with respect to competing benefits and problems. Compared with traditional VALG staging and TNM staging, the nomograms have better prognostic predictive value, which could show better prospects in clinical application. Compared with Qie's^44^ and Wang's^26^ study based on the online databases, our study included routinely available clinical data, relatively comprehensive hematological indicators, detailed treatment regimen and follow‐up information. Nomograms of OS and PFS based on the real‐world population may be more suitable and accurate for predicting the survival of Chinese SCLC patients. Most important of all, we used Pearson correlation coefficient test to ensure that there was no potential collinearity and interaction between each variable so that our nomogram models could have better predictive performances.

There are some limitations in our study. First, this was a retrospective study and certain biases were inevitable. A prospective study is warranted to evaluate the nomogram models in the prognostic prediction of the SCLC patients in the future. Also, the nomograms in this study were established and validated internally which should be validated externally on a larger number of patients at multiple institutions. Second, the NCCN Guidelines for SCLC have approved that the PD‐L1 inhibitors combining chemotherapy could be a new standard first‐line treatment for ES‐SCLC patients, but owing to the inaccessibility of drugs or lack of reimbursement by medical insurance companies in China, only 22 SCLC patients received PD‐L1 inhibitors combined with chemotherapy in this study. Whether these prognostic markers are able to predict the efficacy and prognosis of immunotherapeutic patients remains to be further explored. Third, other clinical information such as history of non‐neoplastic diseases and complications were not recorded in our study which may inevitably lead to a certain bias. Finally, our nomograms were mainly built and validated based on the whole 612 SCLC patients. It is well known that there is a difference in survival rate between patients with ES‐SCLC and LS‐SCLC. These predictors may have some bias between SCLC patients of different VALG stages. In spite of these shortcomings, our nomograms were established based on the large sample real‐world evidence of survival of Chinese SCLC patients, and the nomogram models were found to have better stability and accuracy in validation.

In conclusion, we constructed and validated nomogram to predict OS and PFS for patients with SCLC. The two nomograms outperform the traditional VALG staging system and provide more accurate prediction for the survival of SCLC patients. Inflammatory markers such as CAR, NLR, and AGR should be used widely to identify SCLC patients who could benefit from immunotherapy and better guide clinical treatment. The two proposed survival prognostic models could be helpful to better guide individualized treatment surveillance and decision.

## AUTHOR CONTRIBUTIONS

**Chang Liu:** Writing – original draft (lead). **Bo Jin:** Data curation (equal); supervision (equal); writing – review and editing (equal). **Yunpeng Liu:** Data curation (equal). **Ouyang Juhua:** Writing – original draft (supporting). **Bowen Bao:** Methodology (equal). **Bowen Yang:** Methodology (equal). **Xiuming Liu:** Methodology (equal). **Ping Yu:** Data curation (equal). **Ying Luo:** Data curation (equal). **Shuo Wang:** Data curation (equal). **Zan Teng:** Data curation (equal). **Na Song:** Data curation (equal). **Jinglei Qu:** Data curation (equal). **Jia Zhao:** Data curation (equal). **Ying Chen:** Data curation (equal). **Xiujuan Qu:** Data curation (equal); supervision (equal); writing – review and editing (equal). **Lingyun Zhang:** Data curation (equal); supervision (equal); writing – review and editing (equal).

## FUNDING INFORMATION

This work was supported by High‐level Talent Introduction Support Project (Project No. 36003).

## CONFLICT OF INTEREST STATEMENT

The authors declare no conflict of interest

## ETHICS STATEMENT

Research involving human subjects was reviewed and approved by the Ethics Review Board of the First Hospital of China Medical University (No. [2022] 324). Written informed consent to participate in the study was not required according to national legislation and institutional requirements.

## Data Availability

Raw datasets of this research will be obtained from the corresponding author upon reasonable request.
